# Performance of XL Sizes of Myval Balloon-Expandable Valve in Real-World Patients with Extremely Large Aortic Annuli

**DOI:** 10.3390/medicina62030585

**Published:** 2026-03-20

**Authors:** Kasparas Briedis, Kristina Morkūnaitė, Norvydas Zapustas, Evelina Zarambaitė, Žilvinas Krivickas, Sandra Kmitaitė, Agnė Rimkutė, Klaudija Tvaronavičiūtė, Kamilija Briedė, Urtė Lukauskaitė, Monika Biesevičienė, Tsung-Ying Tsai, Ali Aldujeli, Jurgita Plisienė, Ramūnas Unikas, Remigijus Žaliūnas, Lina Bardauskienė

**Affiliations:** 1 Heart Centre, Lithuanian University of Health Sciences, 44307 Kaunas, Lithuania; 2 Medical Academy, Lithuanian University of Health Sciences, 44307 Kaunas, Lithuania; 3 Boston Scientific, Ballybrit Business Park, H91 Y868 Galway, Ireland; 4 University Hospital Limerick, V94 F858 Limerick, Ireland; 5 Bon Secours Hospital Limerick, V94 892F Limerick, Ireland

**Keywords:** aortic stenosis, TAVI, large aortic annulus, Myval THV

## Abstract

*Background and Objectives:* Transcatheter aortic valve replacement (TAVR) in large aortic annuli poses challenges due to limited valve-size options and increased complication risks. The aim is to evaluate the safety and performance of XL sizes (30.5 mm and 32 mm) of the Myval transcatheter heart valve (THV) for treating patients with severe aortic stenosis and large aortic annuli. *Material and Methods:* This retrospective observational study included consecutive patients undergoing TAVR with XL sizes of the Myval THV between December 2023 and December 2024 at a single centre. During this period, 146 TAVI procedures were performed, of which 15 patients (10.3%) with large aortic annuli (mean systolic annular area 786.5 ± 48.2 mm^2^) received XL valves and were included in the present analysis. Patients were followed up at discharge, 3–6 months, and 1 year. Patient evaluation included echocardiography and clinical assessments following the Valve Academic Research Consortium-3 criteria. *Results:* All patients were male, with a mean age of 79.1 ± 5.9 years. Technical success was achieved in 100% of cases. At discharge, none of the patients had moderate or greater paravalvular leakage (PVL); 11 patients had no PVL, while 1 had trace and 3 had mild PVL. The mean effective orifice area (EOA) improved from 0.75 ± 0.15 cm^2^ at baseline to 2.31 ± 0.21 cm^2^ at discharge (*p* < 0.0001). At the 12-month follow-up, the mean EOA was 2.4 ± 0.3 cm^2^, and no moderate or severe PVL or major adverse clinical outcomes were observed. One patient required a permanent pacemaker implantation due to an atrioventricular block. *Conclusions:* The XL sizes of Myval THV showed both safety and efficacy in patients with large aortic annuli, demonstrating acceptable hemodynamic performance and low complication rates. However, large-scale studies with longer follow-ups are needed to validate these findings in diverse populations.

## 1. Introduction

While transcatheter aortic valve replacement (TAVR) has revolutionised the treatment of severe aortic stenosis (AS), patients with extra-large aortic annulus (diameter ≥ 30 mm) have only one option for TAVR [1,2]. Because of the size limitations of the most widely used transcatheter heart valve (THV) devices until now, such patients suffering from severe AS have not been able to benefit from TAVR [3]. The size of the THV selected for a patient depends on the sizing charts provided by the manufacturer [3]. For example, the self-expanding Evolut R (ER) (Medtronic, Inc., Minneapolis, MN, USA) valve and the balloon-expandable SAPIEN 3 (S3) (Edwards Lifesciences, Irvine, CA, USA) valve are recommended for an annulus up to 683 mm^2^ and an annulus perimeter up to 94.2 mm for the S3 29 mm and the ER 34 mm size, respectively [4]. The size of a patient’s native aortic annulus has a significant impact on TAVR success and subsequent valve performance [4]. An inappropriate size can cause paravalvular leakage (PVL), residual transvalvular gradient (TVG), and other complications, including rupture of the annulus, obstruction of coronary ostia, atrioventricular block, or valve embolisation [1]. Large aortic annuli are associated with higher rates of complications, especially moderate and severe PVL, vascular complications, and permanent pacemaker implantation (PPI). Moderate and severe PVL are predictors of 1-year mortality [4]. Compared to patients with extremely small annuli, those large annuli (area > 683 mm^2^) are more frequently encountered in real-world settings [3]. Besides the risks of PVL and residual transvalvular pressure gradient, THVs small for the annular size can also lead to migration of the valve through the ventricle or aorta [5]. An ideal device-to-annular oversizing of approximately 10–15% (corresponding to a device-to-annulus ratio of 110–115%) is generally recommended [6].

The Myval THV (Meril Life Sciences Pvt. Ltd., Vapi, Gujarat, India) is a new-generation balloon-expandable device approved for the treatment of severe symptomatic native AS in intermediate- and high-risk patients [7]. Myval THV is available in various sizes: conventional (20, 23, 26, and 29 mm), intermediate (21.5, 24.5, and 27.5 mm), and extra-large (30.5 and 32 mm), with a 1.5 mm diameter increment between each nominal device size. The availability of intermediate and extra-large sizes of Myval THV provides an option to implant a THV within the recommended device-to-annular size ratio range [6]. Myval THV series has demonstrated its safety and efficacy in patients with severe symptomatic AS with high-, intermediate-, and low-risk categories and in those with bicuspid anatomy [8,9,10,11,12,13,14,15]. The multi-centre experience of treating extra-large annuli using extra-large sizes of Myval THV have showcased the clinical relevance of the extra-large sizes [7].

We share this case series of 15 patients from a single centre who had large aortic annuli and were implanted with 30.5 and 32 mm Myval THV and followed up until 12 months after TAVR.

## 2. Materials and Methods

This retrospective, observational study analysed data from consecutive patients who underwent TAVR with XL sizes (30.5 and 32 mm) of the Myval THV between December 2023 and December 2024 at a single centre in Lithuania (Hospital of the Lithuanian University of Health Sciences Kaunas Clinics). During this period, a total of 146 TAVI procedures were performed at our institution, of which 15 patients (10.3%) required XL valve sizes due to large annular anatomy and were included in the present analysis.

The study protocol was reviewed and approved by the Bioethics Center of the Lithuanian University of Health Sciences (protocol code 2025-BEC2-0226). Given the retrospective study design and the use of anonymised patient data, the requirement for informed consent was waived. The study was conducted in accordance with the principles of the Declaration of Helsinki.

Pre-procedural evaluation included physical examination, medical history, laboratory investigations, electrocardiogram, transthoracic echocardiography to assess the structure and function of the cardiac valves, and multislice computed tomography to assess the cardiac structure and functions. Post-TAVR, all patients were kept under observation to monitor clinical events and underwent post-procedural electrocardiogram, echocardiogram, and laboratory investigations. Patients were evaluated for procedural success, in-hospital outcomes, and 3-, 6-, and 12-month outcomes based on the Valve Academic Research Consortium-3 (VARC-3) consensus document [16].

**Figure 1:**
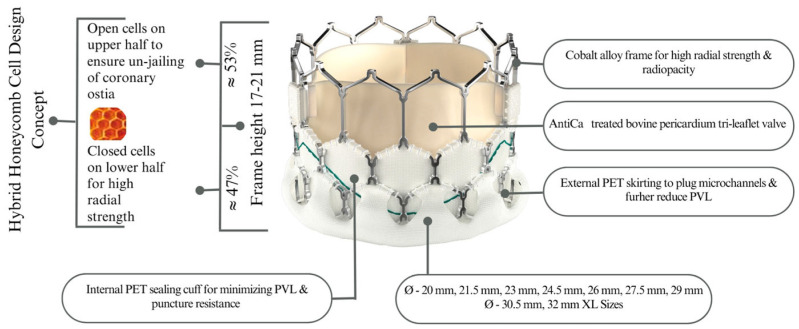
Design of Myval transcatheter heart valve. AntiCa—anti-calcification; mm—millimetre; Ø—diameter; PET—polyethylene terephthalate.

The design features of the Myval THV have been described previously [6]. Briefly, the Myval is a balloon-expandable, bovine pericardial transcatheter heart valve mounted on a cobalt alloy frame and equipped with an external polyethylene terephthalate sealing cuff designed to minimise paravalvular leakage. The valve is available in conventional, intermediate, and extra-large sizes (up to 32 mm), allowing treatment across a wide range of annular anatomies. A schematic representation of the device is shown in Figure 1.

Results are reported as mean ± standard deviation for continuous variables and as number (%) for nominal variables. Continuous variables were presented as mean ± SD in line with the prior TAVR literature reporting hemodynamic outcomes. Given the exploratory nature of this case series and the absence of extreme outliers, parametric analysis was considered appropriate. Hemodynamic variables at different follow-up time points were compared with baseline values using a paired Student’s *t*-test. A *p*-value ≤ 0.05 was considered statistically significant.

## 3. Results

All 15 patients in this case series were male. Their age ranged from 69 to 88 years (mean 79.1 ± 5.9 years). All patients had tricuspid aortic valve morphology. The mean EuroSCORE-II was 3.8%, indicating a moderate risk. Out of 15 patients, 40% belonged to NYHA class II, 46.6% to class III, and 13.3% to class IV. Seven patients had permanent atrial fibrillation at baseline, and four had a PPI. The mean aortic valve area was 0.7 + 0.2 cm^2^, the mean aortic annulus diameter was 2.6 + 0.1 cm, the mean systolic annular aortic area was 786.5 + 48.2 mm^2^, and the mean effective orifice area (EOA) was 0.75 + 0.15 cm^2^. Therefore, none of the available TAVR devices in the market were suitable for this patient population. The baseline characteristics are shown in Table 1.

All patients underwent transfemoral TAVR under conscious sedation, and the mean duration of the procedure was 37.7 min. Eleven patients required pre-dilatation, which was performed using the Mammoth balloon catheter (Meril Life Sciences Pvt. Ltd., Vapi, Gujarat, India); of these, eight patients required a size 25 mm and three required a size 23 mm pre-dilatation balloon. Ten patients required the 32 mm Myval THV, while five required 30.5 mm. Only one patient required post-dilatation due to moderate PVL, which was performed using a 25 mm Mammoth balloon. Technical and procedural success was achieved in all cases, with the THV successfully deployed at the appropriate anatomical location. No patient required conversion to open surgery. One patient had a new onset left bundle branch block (LBBB) after THV implantation. There were no other major or minor complications associated with the procedure. The procedural details are shown in Table 2.

At the time of discharge, the mean EOA was 2.31 + 0.2 cm^2^. Of the 15 patients, none of the patients had moderate or greater PVL, 11 had no PVL, 1 had a trace PVL, and 3 had a mild PVL. One patient required a new single-lead ventricular demand with ventricular backup and inhibited (VVI) PPI due to a complete AV block, on day 2 post-TAVI. The outcomes at the time of discharge are shown in Table 3.

The outcomes at 3–6 months and 12-month follow-up are shown in Table 4. All patients retained their baseline condition of sinus rhythm or permanent atrial fibrillation at both time points, with no conduction disturbances. The mean EOA was 2.3 + 0.2 and 2.4 + 0.3 cm^2^, respectively. At 3–6 months and 12 months, none of the patients had moderate or greater PVL. There were no major or minor adverse clinical outcomes.

**Figure 2:**
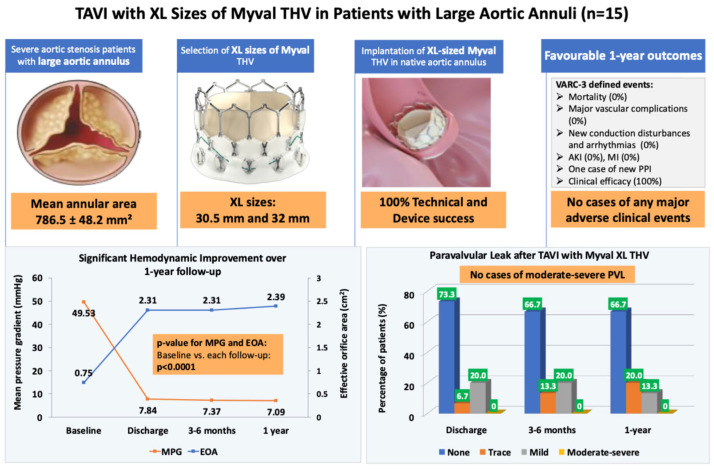
Central illustration. AKI—acute kidney injury; EOA—effective orifice area; MI—myocardial infarction; MPG—mean pressure gradient; PPI—permanent pacemaker implantation; PVL—paravalvular leak; TAVI—transcatheter aortic valve implantation; THV—transcatheter heart valve; VARC-3—valve academic research consortium-3; XL—extra-large.

Procedural success, valve performance, and short- to mid-term clinical outcomes are summarised in Figure 2 (Central Illustration).

## 4. Discussion

Our case series of 15 patients demonstrates the successful outcomes with Myval THV in patients with large aortic annuli. Only one patient (6.6%) required post-dilatation after implantation, indicating that the THV sizes were appropriate for most patients, and only one patient (6.6%) developed a new-onset LBBB after TAVR. Overall, 26.67% of patients had mild or trace PVL, while none had moderate or severe PVL. One patient (6.6%) required permanent pacemaker implantation due to complete atrioventricular block during the index hospitalisation (on day 2 after TAVR), with no additional PPI events observed during the 12-month follow-up, while none of the patients had moderate or greater PVL. There was no mortality or any other adverse outcome. Few studies have reported the outcomes of TAVR in patients with large aortic annuli. Research on patients with very large aortic annulus dimensions (aortic area > 740 mm^2^) is scarce and mainly consists of small case series [7]. A previous study evaluating earlier-generation self-expanding supra-annular transcatheter bioprostheses demonstrated that below-range device oversizing was linked to a relatively high rate of moderate or greater paravalvular aortic regurgitation (15.3%) [17]. Tang et al. investigated the outcomes of the S3 valve in 74 patients with extra-large annuli. The mean area was 721 ± 38 mm^2^ (range: 684 to 852 mm^2^). Post-dilatation was required in 32% of patients, new LBBB occurred in 17%, and 6.3% required new PPI. Thirty-day echocardiography showed mild PVL in 22.3% and moderate PVL in 6.9% [18]. In another study of S3 valve in 30 patients with large annuli (737.3 ± 54.7 mm^2^), Miyasaka et al. reported at least mild PVL in 43.3% of patients, moderate or greater in 3.3%, and 1-year mortality of 10.0% [3]. In the literature, the incidence of mild or worse PVL ranges from 19.7% to 48.5% among patients who were implanted with the S3 THV [3]. The Society of Thoracic Surgeons/American College of Cardiology Transcatheter Valve Therapy Registry reported the TAVR outcomes in 1096 patients with large annuli (diameter 26 to 34 mm) using 34 mm Evolut R/PRO+ valves [2]. Patients with annular diameters of 30–34 mm demonstrated less favourable outcomes. Moderate-to-severe PVL was seen in 6.4% of patients. At 1-year follow-up, mortality was 19.6%, aortic valve reintervention rate was 2.1%, and valve-related readmission rates were 3.2%. By 1 year, 27.7% of patients had undergone new permanent pacemaker or implantable cardioverter-defibrillator implantation. The authors recommended that the use of the Evolut R/PRO+ 34 mm valve in patients with large annuli be regarded as off-label, with alternative treatment strategies considered individually [2]. More recently, Hof et al. reported outcomes and device performance of the S3 29 mm and ER 34 mm THVs in a cohort of patients with extra-large aortic annuli (730.4 ± 53.9 mm^2^). Technical success was 96.5%, device success was 82.3%, and moderate PVL occurred in 8.9% at 30 days, while the rate of new PPI was 7% [4]. Notably, most of these studies have used the S3 valve or 34 mm Evolut R/PRO+ valves in patients with large aortic annuli as an “off-label” indication by overexpansion of the valves. In a bench-testing study, S3 prostheses with diameters of 23, 26, and 29 mm were overexpanded using balloons 1, 2, and 3 mm larger than the recommended diameter. Visible restriction of the valve leaflets was observed, particularly in the smaller valves. Following maximal overexpansion of the 26 mm valve, a leaflet tear occurred. High-speed video analysis demonstrated impaired leaflet motion in the 23 and 26 mm valves, and hydrodynamic testing revealed a regurgitation fraction above accepted international standards for the 23 and 26 mm valves. The study concluded that excessive overexpansion may adversely affect hydrodynamic performance and may be associated with acute leaflet failure and diminished durability [19]. Sellers et al. evaluated the effect of valve overexpansion on leaflet ultrastructure in an ex vivo model across valve sizes of 23, 26, and 29 mm. Overexpanded valves demonstrated structural leaflet damage, thinning, and increased tissue density within the leaflet matrix relative to nominally expanded controls [20].

Annular dimensions falling within ±15% of the upper or lower sizing limits for a given self-expandable THV are commonly referred to as a “borderline annulus” [1]. Yildirim et al. found that outcomes were comparable between balloon-expandable and self-expandable valves when annulus sizes were within borderline zones. In their study, the frequency of PVL (mild, mild to moderate, and moderate) was 40.2%, 11.8%, and 2.9% in patients with annuli in borderline zones [1]. A unique feature of the Myval system is its availability in nine different sizes, spaced 1.5 mm apart, ranging from 20 to 32 mm, enabling treatment across a wide spectrum of annular dimensions [21]. The availability of intermediate-size Myval valves may be particularly beneficial for patients with annuli falling within borderline sizing ranges [1]. Further, the 32 mm Myval THV is currently the largest available aortic THV, with recommended sizing for annular areas of 700–840 mm^2^, a range not covered by other available THVs [7]. No published studies have included patients with an annular area as large as 840 mm^2^; however, Holzamer et al. reported promising initial results in patients with very large annular anatomy (mean area 765.5 mm^2^) treated with the 32 mm Myval transcatheter heart valve, demonstrating acceptable procedural safety and early hemodynamic performance [7]. Moreover, among 2219 consecutive patients screened for TAVR at a Central European centre, only 0.27% had annular dimensions beyond the coverage of the 32 mm Myval device [7]. The first prospective trial cohort to include patients with extra-large anatomy is the nested Myval XL registry within the ongoing LANDMARK trial (ClinicalTrials.gov NCT04275726). Participants in this trial will undergo follow-up for 10 years after TAVR [22].

There are certain limitations associated with our study. The first is that the sample size is only 15 patients. In addition, newer iterations of the Myval platform, such as Myval Octapro and Octapro+, have been introduced in recent years. Although the XL valve sizes used in this study retain the same nominal diameters and fundamental structural characteristics, procedural behaviour and implantation characteristics may differ with newer device generations. Another limitation is that all patients had tricuspid anatomy. The higher challenges with TAVR in bicuspid anatomy are well-known. The third limitation is the relatively short duration of follow-up. However, the strength of our study is that this is possibly the first series reporting outcomes in annuli of 786.45 ± 48.23 mm^2^. Our findings need to be validated in larger cohorts, including patients with bicuspid anatomy and with longer follow-up duration.

## 5. Conclusions

Extra-large sizes of the Myval THV provide a safe and effective treatment option for patients with severe AS and large aortic annuli. The procedure demonstrated satisfactory hemodynamic performance with minimal rates of PVL through 12-month follow-up. These promising outcomes suggest that the Myval THV XL sizes may address the unmet need for transcatheter valve solutions in patients with large aortic annuli, offering favourable short- and mid-term results. Further studies with larger cohorts and long-term follow-up are necessary to confirm these findings.

## Figures and Tables

**Table 1:** Baseline characteristics.

Baseline Characteristics	*n* = 15
Age	79.13 ± 5.91
Sex	
Male	15 (100.00)
BMI, kg/m^2^	27.34 ± 2.94
BSA, m^2^	2.1 ± 0.14 (*n* = 15)
EuroSCORE-II, %	3.84 ± 0.73
NYHA	
I	0 (0.00)
II	6 (40.00)
III	7 (46.67)
IV	2 (13.33)
6 min Walk test, m	264.53 ± 80.34
ECG findings	
Permanent atrial fibrillation	7 (46.67)
Normal Sinus Rhythm	8 (53.33)
NT-proBNP, ng/L	576.45 ± 305.82
eGFR, mL/min/1.73 m^2^	56.13 ± 13.61
Previous CABG	1 (6.67)
Previous PCI	11 (73.33)
Previous MI	4 (26.67)
Previous PPI	4 (26.67)
Previous stroke	2 (13.33)
COPD	3 (20.00)
Arterial hypertension	14 (93.33)
Dyslipidaemia	14 (93.33)
Diabetes mellitus	2 (13.33)
**Baseline Cardiac Computed Tomography (TAVI protocol)**	
Systolic annular aortic perimeter, mm	99.24 ± 4.24
Perimeter-derived annular diameter, mm	31.59 ± 1.35
Systolic annular aortic area, mm^2^	786.45 ± 48.23
Area-derived annular diameter, mm	31.65 ± 0.95
Aortic annulus angle, (degree)	50.67 ± 9.48
Perimeter-derived LVOT diameter, mm	31.43 ± 1.24
Area-derived LVOT diameter, mm	31.62 ± 1.17
SoV diameters—right coronary sinus, mm	36.38 ± 1.54
SoV diameters—left coronary sinus, mm	36.41 ± 2.05
SoV diameters—non-coronary sinus, mm	37.13 ± 1.73
Coronary height—right coronary artery, mm	17.1 ± 3.28
Coronary height—left coronary artery, mm	15.27 ± 2.25
Sinotubular junction diameter, mm	33.02 ± 1.42
Average ascending aorta diameter, mm	38.01 ± 2.19
Perpendicular Plane—Calcium Quantification, mm^3^	1419.53 ± 549.9
Annular/Subannular calcification qualitative	
None	4 (26.67)
Mild	5 (33.33)
Moderate	5 (33.33)
Severe	1 (6.67)
Left common iliac average diameter, mm	11.03 ± 1.37
Left external iliac average diameter, mm	9.09 ± 0.87
Left femoral average diameter, mm	8.78 ± 0.9
Calcification	
None	2 (13.33)
Mild	11 (73.33)
Moderate	2 (13.33)
Right common iliac average diameter, mm	11.15 ± 1.21
Right external iliac average diameter, mm	9.4 ± 1.08
Right femoral average diameter, mm	9.07 ± 0.87
Calcification	
None	2 (13.33)
Mild	10 (66.67)
Moderate	3 (20.00)
**2D echocardiography (TTE)**	
EOA, cm^2^	0.75 ± 0.15
iEOA, cm^2^/m^2^	0.36 ± 0.08
Aortic annulus diameter, cm	2.6 ± 0.13
Mean AV gradient, mmHg	49.53 ± 8.27
Max AV jet velocity, m/sec	4.44 ± 0.24
Peak AV gradient, mmHg	80.39 ± 12.07
Aortic regurgitation	
None	1 (6.67)
Mild	10 (66.67)
Mild-Moderate	2 (13.33)
Moderate	2 (13.33)
Tri-leaflet aortic valve	15 (100.00)
Pulmonary artery pressure, mmHg	42.33 ± 8.62
LVEDD, cm	4.83 ± 0.26
Left ventricular ejection fraction, %	51.87 ± 3.8
TAPSE, mm	21.47 ± 2.64
Peak tricuspid regurgitation systolic velocity, cm/sec	295.8 ± 27.26
Mitral valve moderate/severe regurgitation	1 (6.67)
Tricuspid valve moderate/severe regurgitation	3 (20.00)
Pulmonary valve moderate/severe regurgitation	0 (0.00)

Values are shown as mean ± standard deviation or *n* (%). AV—aortic valve; BMI—body mass index; BSA—body surface area; CABG—coronary artery bypass graft; COPD—chronic obstructive pulmonary disease; ECG—electrocardiogram; eGFR—estimated glomerular filtration rate; EOA—effective orifice area; iEOA—indexed effective orifice area; LVEDD—left ventricular end-diastolic diameter; LVOT—left ventricular outflow tract; MI—myocardial infarction; NT-proBNP—n-terminal pro-B-type natriuretic peptide; NYHA—New York heart association; PCI—percutaneous coronary intervention; PPI—permanent pacemaker implantation; PVL—paravalvular leakage; SoV—sinus of valsalva; TAPSE—tricuspid annular plane systolic excursion; TAVI—transcatheter aortic valve implantation; TTE—transthoracic echocardiogram.

**Table 2:** Procedural details.

Procedural Details	*n* = 15
Procedure time (skin to skin), min	37.72 ± 4.75
Arterial access site	
TF Left	2 (13.33)
TF Right	13 (86.67)
Pre-dilation	11 (73.33)
Myval XL sizes implanted, mm	
30.5	5 (33.33)
32	10 (66.67)
Post-dilation	1 (6.67)
Correct positioning of the valve in the appropriate anatomical location	15 (100.0)
Successful delivery and deployment of valve	15 (100.0)
Conversion from TAVI to surgery	0 (0.00)
Valve embolisation	0 (0.00)
Major/minor vascular complications (VARC-3)	0 (0.00)
Access-related non-vascular major/minor complications (VARC-3)	0 (0.00)
Overt bleeding complications (VARC-3)	0 (0.00)
Cardiac structural major/minor complications (VARC-3)	0 (0.00)
Any other acute procedural and technical valve-related complications (VARC-3)	0 (0.00)
Any new conduction disturbances and arrhythmias (VARC-3)	1 (6.67)
Myocardial infarction (VARC-3)	0 (0.00)
Any Concomitant Procedures	0 (0.00)
Technical Success (VARC-3 Composite Endpoint)	15 (100.0)

Values are shown as mean ± standard deviation or *n* (%). TAVI—transcatheter aortic valve implantation; TF—transfemoral; VARC-3—valve academic research consortium-3.

**Table 3:** Outcomes at discharge.

Clinical Safety Outcomes		Discharge
Mortality (VARC-3)		0 (0.00)
New neurological events (VARC-3)		0 (0.00)
New overt bleeding or transfusions (VARC-3)		0 (0.00)
New major/minor vascular and access-related complications (VARC-3)		0 (0.00)
New major/minor cardiac structural complications (VARC-3)		0 (0.00)
New other acute procedural and technical valve-related complications (VARC-3)		0 (0.00)
New conduction disturbances and arrhythmias requiring PPI (VARC-3)		1 (6.67)
New myocardial infarction (VARC-3)		0 (0.00)
Acute kidney injury (VARC-3)		0 (0.00)
Aortic prosthesis valve dysfunction (VARC-3)		0 (0.00)
Bioprosthesis valve deterioration (VARC-3)		0 (0.00)
Clinically significant valve thrombosis (VARC-3)		0 (0.00)
**Device Success. Composite Endpoints at discharge based on VARC-3**		
Yes		15 (100.0)
No		0 (0.00)
**2D echocardiography (TTE)**	**Discharge**	***p*** **-value** **(Baseline vs. Discharge)**
BSA, m^2^	2.1 ± 0.14	
EOA, cm^2^	2.31 ± 0.21	<0.0001
iEOA, cm^2^/m^2^	1.1 ± 0.11	
Aortic annulus diameter, cm	-	
Mean AV gradient, mmHg	7.84 ± 2.33	<0.0001
Max AV jet velocity, m/sec	1.75 ± 0.22	
Peak AV gradient, mmHg	14.21 ± 5.25	
PVL, degree (trace, mild, moderate, severe)		
None	11 (73.33)	
Trace	1 (6.67)	
Mild	3 (20.00)	
Pulmonary artery pressure, mmHg	43 ± 7.27	
LVEDD, cm	4.79 ± 0.3	
Left ventricular ejection fraction, %	51.87 ± 3.8	
TAPSE, mm	21.27 ± 1.75	
Peak tricuspid regurgitation systolic velocity, cm/sec	288.67 ± 26.96	
Mitral valve moderate/severe regurgitation	1 (6.67)	
Tricuspid valve moderate/severe regurgitation	3 (20.00)	
Pulmonary valve moderate/severe regurgitation	0 (0.00)	

Values are shown as mean ± standard deviation or *n* (%). AV—aortic valve; AVB—atrioventricular block; BSA—body surface area; EOA—effective orifice area; F/U—follow-up; iEOA—indexed effective orifice area; LVEDD—left ventricular end-diastolic diameter; NYHA—New York heart association; PPI—permanent pacemaker implantation; PVL—paravalvular leakage; TAPSE—tricuspid annular plane systolic excursion; TAVI—transcatheter aortic valve implantation; VARC-3—valve academic research consortium-3; VVI—single-lead ventricular demand with ventricular backup and inhibited PPI.

**Table 4:** Outcomes at 3–6 months and 12 months.

Clinical Safety Outcomes	3–6 Months Follow-Up	12-Month Follow-Up
Mortality (VARC-3)	0 (0.00)	0 (0.00)
Neurological events (VARC-3)	0 (0.00)	0 (0.00)
Overt bleeding or transfusions (VARC-3)	0 (0.00)	0 (0.00)
Major/minor vascular and access-related complications (VARC-3)	0 (0.00)	0 (0.00)
New major/minor cardiac structural complications (VARC-3)	0 (0.00)	0 (0.00)
New other acute procedural and technical valve-related complications (VARC-3)	0 (0.00)	0 (0.00)
New conduction disturbances and arrhythmias (VARC-3)	0 (0.00)	0 (0.00)
New myocardial infarction (VARC-3)	0 (0.00)	0 (0.00)
Aortic prosthesis valve dysfunction (VARC-3)	0 (0.00)	0 (0.00)
Bioprosthesis valve deterioration (VARC-3)	0 (0.00)	0 (0.00)
Clinically significant valve thrombosis (VARC-3)	0 (0.00)	0 (0.00)
**Early Safety Composite Endpoints based on VARC-3 (up to 6 months)**		
Achieved all early safety composite outcomes based on VARC-3	14 (93.33)	--
New PPI post-TAVI due to complete AVB (index hospitalisation)	1 (6.67)	--
**Clinical Efficacy Composite Endpoints based on VARC-3 (at 12-month F/U)**		
Achieved all clinical efficacy composite outcomes based on VARC-3	--	15 (100.0)
**2D echocardiography (TTE)**	**3–6 Months** **Follow-up**	**12-Month** **Follow-up**
BSA, m^2^	2.09 ± 0.13	2.08 ± 0.1
EOA, cm^2^	2.31 ± 0.22 (*p* < 0.0001 vs. baseline)	2.39 ± 0.26 (*p* < 0.0001 vs. baseline)
iEOA, cm^2^/m^2^	1.11 ± 0.11	1.15 ± 0.12
Mean AV gradient, mmHg	7.37 ± 1.74 (*p* < 0.0001 vs. baseline)	7.09 ± 1.78 (*p* < 0.0001 vs. baseline)
Max AV jet velocity, m/sec	1.65 ± 0.16	1.64 ± 0.15
Peak AV gradient, mmHg	13.95 ± 3.9	14.09 ± 3.21
PVL, degree (trace, mild, moderate, severe)		
None	10 (66.67)	10 (66.67)
Trace	2 (13.33)	3 (20.00)
Mild	3 (20.00)	2 (13.33)
Pulmonary artery pressure, mmHg	41.67 ± 5.56	40.67 ± 5.3
LVEDD, cm	4.77 ± 0.24	4.86 ± 0.28
Left ventricular ejection fraction, %	52.33 ± 3.72	52.53 ± 3.36
TAPSE, mm	21 ± 1.31	21.53 ± 1.64
Peak tricuspid regurgitation systolic velocity, cm/sec	280.67 ± 23.74	277.33 ± 27.12
Mitral valve moderate/severe regurgitation	1 (6.67)	1 (6.67)
Tricuspid valve moderate/severe regurgitation	3 (20.00)	3 (20.00)

Values are shown as mean ± standard deviation or *n* (%). AV—aortic valve; AVB—atrioventricular block; BSA—body surface area; EOA—effective orifice area; F/U—follow-up; iEOA—indexed effective orifice area; LVEDD—left ventricular end-diastolic diameter; NYHA—New York heart association; PPI—permanent pacemaker implantation; PVL—paravalvular leakage; TAPSE—tricuspid annular plane systolic excursion; TAVI—transcatheter aortic valve implantation; VARC-3—valve academic research consortium-3; VVI—single-lead ventricular demand with ventricular backup and inhibited PPI.

## Data Availability

The datasets generated and/or analysed during this single-centre retrospective study are available from the corresponding author upon reasonable request. The data are not publicly available due to ethical and institutional restrictions related to the use of anonymized patient data.

## Abbreviations

The following abbreviations are used in this manuscript:

A KI	acute kidney injury
AS	aortic stenosis
AV	aortic valve
AVB	atrioventricular block
BMI	body mass index
BSA	body surface area
CABG	coronary artery bypass graft
COPD	chronic obstructive pulmonary disease
ECG	electrocardiogram
eGFR	estimated glomerular filtration rate
EOA	effective orifice area
F/U	follow-up
iEOA	indexed effective orifice area
LBBB	left bundle branch block
LVEDD	left ventricular end-diastolic diameter
LVOT	left ventricular outflow tract
MI	myocardial infarction
MPG	mean pressure gradient
NT-proBNP	N-terminal pro–B-type natriuretic peptide
NYHA	New York Heart Association
PCI	percutaneous coronary intervention
PET	polyethylene terephthalate
PPI	permanent pacemaker implantation
PVL	paravalvular leak
S3	SAPIEN 3
SoV	sinus of Valsalva
TAVI	transcatheter aortic valve implantation
TAVR	transcatheter aortic valve replacement
TAPSE	tricuspid annular plane systolic excursion
TF	transfemoral
THV	transcatheter heart valve
TTE	transthoracic echocardiography
TVG	transvalvular gradient
VARC-3	Valve Academic Research Consortium-3
VVI	ventricular demand with ventricular backup and inhibited pacing
XL	extra-large
